# Repertoire analysis of γδ T cells in the chicken enables functional annotation of the genomic region revealing highly variable pan-tissue TCR gamma V gene usage as well as identifying public and private repertoires

**DOI:** 10.1186/s12864-021-08036-9

**Published:** 2021-10-06

**Authors:** Robert Dixon, Stephen G. Preston, Stefan Dascalu, Patrik G. Flammer, Steven R. Fiddaman, Kirstie McLoughlin, Amy Boyd, Jiri Volf, Ivan Rychlik, Michael B. Bonsall, Bernd Kaspers, Adrian L. Smith

**Affiliations:** 1 Department of Zoology, University of Oxford, Oxford, UK; 2 The Pirbright Institute, Ash Road, Pirbright, Woking, Surrey United Kingdom; 3 Veterinary Research Institute, Brno, Czech Republic; 4 Veterinary Faculty, Ludwig Maximillians University, Planegg, Germany

**Keywords:** Chicken, Gamma, Repertoire, T cell, TCR, Sequencing, Locus

## Abstract

**Background:**

Despite increasing interest in γδ T cells and their non-classical behaviour, most studies focus on animals with low numbers of circulating γδ T cells, such as mice and humans. Arguably, γδ T cell functions might be more prominent in chickens where these cells form a higher proportion of the circulatory T cell compartment. The TCR repertoire defines different subsets of γδ T cells, and such analysis is facilitated by well-annotated TCR loci. γδ T cells are considered at the cusp of innate and adaptive immunity but most functions have been identified in γδ low species. A deeper understanding of TCR repertoire biology in γδ high and γδ low animals is critical for defining the evolution of the function of γδ T cells. Repertoire dynamics will reveal populations that can be classified as innate-like or adaptive-like as well as those that straddle this definition.

**Results:**

Here, a recent discrepancy in the structure of the chicken TCR gamma locus is resolved, demonstrating that tandem duplication events have shaped the evolution of this locus. Importantly, repertoire sequencing revealed large differences in the usage of individual TRGV genes, a pattern conserved across multiple tissues, including thymus, spleen and the gut. A single TRGV gene, TRGV3.3, with a highly diverse private CDR3 repertoire dominated every tissue in all birds. TRGV usage patterns were partly explained by the TRGV-associated recombination signal sequences. Public CDR3 clonotypes represented varying proportions of the repertoire of TCRs utilising different TRGVs, with one TRGV dominated by super-public clones present in all birds.

**Conclusions:**

The application of repertoire analysis enabled functional annotation of the TCRG locus in a species with a high circulating γδ phenotype. This revealed variable usage of TCRGV genes across multiple tissues, a pattern quite different to that found in γδ low species (human and mouse). Defining the repertoire biology of avian γδ T cells will be key to understanding the evolution and functional diversity of these enigmatic lymphocytes in an animal that is numerically more reliant on them. Practically, this will reveal novel ways in which these cells can be exploited to improve health in medical and veterinary contexts.

**Supplementary Information:**

The online version contains supplementary material available at 10.1186/s12864-021-08036-9.

## Introduction

The adaptive immune system of jawed vertebrates functions through the production of antibodies by B lymphocytes, combined with the cytotoxic, helper and regulatory functions of T lymphocytes. T lymphocytes can be divided into two lineages according to their expression of either the αβ or γδ form of the T cell receptor (TCR), and the appearance of these lineages coincides with the emergence of the jawed vertebrates [1, 2]. The function of TCRαβ+ T cells is well defined in many animal species, with most αβ T cells recognizing peptides presented on the surfaces of cells in the context of MHC class I or II. In contrast, TCRγδ+ T cells are much less well understood, although a range of stimulatory ligands have been described [3], and they have been shown to have an array of cytotoxic and cytokine based effector functions [4]. At least some populations of γδ T cells are considered part of the innate immune system, operating in the early phases of the host’s response. These γδ T cell subsets have very restricted TCR diversity, which is characteristic of an innate-like response [5, 6]. Other γδ T cell populations have diverse CDR3 repertoires, as are seen in classical αβ T cells, more characteristic of the adaptive immune system. Further characterization of the TCR repertoire of γδ T cell populations will be important in exploring whether these populations function in innate immunity, adaptive immunity or both.

Most information on the biology of γδ T cells derives from research on human blood or rodent tissues. Whilst providing a solid foundation for understanding the role of γδ T cells in immune responses, it is noteworthy that some of the aspects of their biology are not shared between humans and rodents, such as the population of Dendritic Epidermal T Cells (DETCs) which express an invariant TCR and are located in the skin of mice [7]. Moreover, humans and rodents are both considered γδ low animals whereas many others, including chickens, are considered γδ high animals [8]. γδ low animals are loosely characterised as having a peripheral blood T cell compartment which consists of less than 10% γδ T cells, the remainder being αβ T cells. This group includes mice (0.5–10%), humans (0.5–10%) [9] and dogs (2.5%) [10]. Conversely, γδ high animals have a circulatory T cell compartment comprised of greater than 10% γδ T cells. This group includes various domesticated species such as chickens (15–50%) [11], cattle (20–40%) [12], pigs (30%) [13] and goats (5–20%) [14, 15]. In both γδ high and γδ low animals, the proportions of γδ T cells are greatly enriched in some tissues, particularly at epithelial sites such as the intestine. To fully appreciate the biology of γδ T cells it is important to consider these lymphocytes in a range of different animals.

Typical γδ T cell functions include; rapid response to pathogens [16, 17], regulation of enterocyte turnover [18], protection against epithelial tumours [19], and limitation of inflammatory damage [20]. γδ T cells are thought to be particularly important for immunity in early life [17, 21]. In birds and mammals, γδ T cells are the first T cells to develop in the thymus [22, 23], and in mice these early γδ T cells migrate from the thymus in discrete waves, each expressing different T Receptor Gamma Variable (TRGV) genes and homing to particular tissues to perform discrete functions [5, 24]. The chicken is a γδ high species in which our knowledge of the response and function of these cells is limited. What is clear is that chicken γδ T cells are heterogeneous and can produce a variety of cytokines and interferons, as well as displaying cytotoxicity [25, 26], and responding to infectious challenge [27, 28] or changes in hormone levels [29]. As T cell specificity is determined by the TCR, advances in high throughput next generation sequencing (NGS) have enabled their biology to be studied in previously unattainable detail using TCR repertoire sequencing.

Accurate description of the clonal repertoire of TCR gamma requires a correctly assembled and annotated genomic locus. Early iterations of the chicken genome did not accurately depict the structure or content of the gamma locus, presumably due to the repetitive nature of the genes and the difficulties this poses to assembly. Although the most recent genome build (galGal6a) is a vast improvement, the sequence and structure of the TCR gamma locus is different to that proposed recently by Liu et al. 2020 [30], based upon BAC insert resequencing. Here, we aim to resolve this discrepancy and add additional annotation that highlights the duplication-based evolution of this locus. Using a 5’RACE based parallel sequencing approach for assessment of repertoire, we show that most TRGV genes are utilised, but that some TRGVs are used much more commonly than others. The proportional usage of each TCRV gene is partially predicted by the sequence of the recombination signal sequence (RSS) but other factors are clearly involved. Furthermore, the chicken TCR gamma repertoire includes substantial portions of highly public CDR3 sequences as well as a large private (individual-specific) repertoire.

## Methods

### Tissue collection

Birds were hatched and reared in dedicated facilities. The ISA Brown birds (*n* = 8) were sacrificed at days 49 post hatch and the PA12 White Leghorns (*n* = 5) at 55 days post hatch and their tissues harvested. Tissue segments (1–2 cm^3^) taken from the ISA Brown birds were Thymus, Spleen, Duodenum, Jejunum, Ileum, Caecum and Colon, and tissues taken from the PA12 White Leghorn birds were Spleen, Bursa, Jejunum, Caecum and Colon. The tissues were processed and placed in RNAlater (Sigma-Aldrich) according to manufacturer’s instructions and stored at − 80 °C.

### Restriction endonuclease digest

Chicken genomic DNA was extracted from spleen samples from PA12 White Leghorn birds (55 days old) using the AllPrep Micro Kit (Qiagen). The samples were homogenised by bead beating and processed according to manufacturer’s instructions. A PCR master mix was then made up using 17.25 μl Nuclease Free H_2_O (Qiagen), 5 μl High Fidelity Buffer (NEB), 0.5 μl 10 mM dNTPs (NEB), 0.25 μl Phusion (NEB), 0.5 μl DrdI_F1/2/3, 0.5 μl DrdI_R1/2/3 and 1 μl genomic DNA. Amplification was performed using the following thermocycler conditions. 1 min at 98 °C, followed by 35 cycles of 15 s at 98 °C, 15 s at 65 °C and 30 s at 72 °C followed by a final extension of 2 mins at 72 °C and a 4 °C holding temperature. All primers are shown in Additional File 1. PCR products were then digested and added to a reaction mix with 5 μl 10X rCutSmart Buffer (NEB) and 1 μl 5 U/μl DrdI, which was then made up to 50 μl by addition of Nuclease Free Water (Qiagen). This reaction mix was then incubated at 37 °C overnight and the resulting products run on a 1.5% agarose gel.

### Gamma locus annotation

TRGV genes were identified by performing BLAST searches using gene sequences previously annotated in the galGal6a genome. BLAST searches used id = 0.95 and evalue = 0.001. These prospective TRGV genes were then confirmed by: (i) Existence of a plausible Restriction Signal-Sequence (RSS) as assessed by Recombination Information Content (RIC) score using RSSsite [31]; (ii) The InterPro protein prediction tool, which was used to ensure the sequence corresponded to an Ig-like like motif [32, 33]. To ensure that no TRGV genes had been missed by this search strategy, the sequences obtained from UPA sequencing were realigned to the gamma locus using Bowtie2 with default settings [34] and the results visualised using SeqMonk [35]. TRGV gene families were assigned using the Ig-like domain predicted by InterPro. Using the International ImMunoGeneTics Information System (IMGT) [36] subgroup concept, which requires 75% sequence identity at the nucleotide level [37], TRGV gene families were assigned. TRGV genes within these families were numbered according to their position in the locus, from distal to proximal with respect to the TRGC gene. Sequence identity was determined by the following formula

$$ 100\times \frac{Identical\ Positions}{Length\ of\ Shorter\ Sequence} $$

### Phylogenetic tree construction

Sequences were aligned using MUSCLE [38] with a gap opening penalty of − 100 and a gap extension penalty of − 10 with the overhanging ends trimmed off. The most appropriate nucleotide evolution model was selected from a table of potential models by determining the highest log-likelihood and lowest AIC values [39]. A distance matrix was calculated and used to create an initial phylogenetic tree using the neighbour joining method [40]. This tree was used as a starting point for maximum likelihood computation and bootstrap values calculated for each branch using 1000 replicates.

### RNA extraction

Tissue samples were defrosted and small subsections taken using a sterile scalpel. These sections were then processed with the AllPrep Micro Kit (Qiagen) as previously described for ‘Restriction endonuclease digestion’. Extracted RNA was assayed for concentration and integrity using the RNA ScreenTape System (Agilent). The SMARTer™ PCR cDNA Synthesis Kit (Clontech) was then used according to manufacturer’s instructions, to convert the RNA to cDNA incorporating a known universal sequence at the 5′ end.

### 5′ RACE cDNA synthesis

5′ Rapid amplification of cDNA ends (RACE) was used to generate cDNA from RNA using the Takara Bio SMARTer reverse transcriptase kit. Master mix was made up of 2 μl 5x First Strand Buffer (Takara Bio), 1 μl 20 mM DTT, 1 μl 10 mM dNTPs (Takara), 1 μl 12 μM SMARTer IIA oligo, 0.5 μl 20 U/μl RNase Inhibitor (Takara) and 1 μl 100 U/μl SMARTScribe Reverse Transcriptase. 1 μl 5′-CDS Primer A was then added to 2.75 μl of each sample RNA and incubated at 72 °C for 3 min, then cooled to 4 °C for 2 min. The RNA and CDS primer was then added to the master mix and incubated at 42 °C for 90 mins then 70 °C for 10 mins in a Thermocycler. 20 μl Tris-EDTA was then added to the resulting mix and the samples stored at − 20 °C.

### Universal UPA repertoire PCRs

PCR reactions were set up in separate triplicate repeats for each sample using a master mix made up of 16.75 μl Nuclease Free Water (Qiagen), 5 μl High Fidelity Buffer (NEB), 0.5 μl 10 mM dNTPs (NEB), 0.5 μl 10 μM UPA short primer, 0.5 μl 2 μM UPA long primer, 0.5 μl 10 μM Barcoded Chicken TCR gamma primer, 0.25 μl Phusion (NEB) and 1 μl cDNA template. Amplification was performed using the following thermocycler conditions. 1 min at 98 °C, followed by 5 cycles of 15 s at 98 °C and 30 s at 72 °C followed by 5 cycles of 15 s at 98 °C, 15 s at 70 °C and 30 s at 72 °C, followed by 30 cycles of 15 s at 98 °C, 15 s at 62 °C and 30 s at 72 °C with a final extension of 2 mins at 72 °C and a 4 °C holding temperature. All primers are shown in Additional File 1.

### Illumina library preparation

PCR products were combined according to their relative concentrations as estimated from an agarose gel. The resulting mix was then run on a separate gel and extracted using the QIAquick Gel Extraction Kit (Qiagen) according to manufacturer’s instructions to remove contaminants. The products were prepared for Illumina sequencing using the NEBNext® Ultra™ DNA Library Prep Kit for Illumina (NEB) and the subsequent concentration of the libraries assayed using the NEBNext® Library Quant Kit for Illumina® (NEB). The resulting libraries were then blended and sequenced on the MiSeq using the MiSeq System Denature and Dilute Libraries kit (Illumina).

### Sequence processing

The data collected from the MiSeq run was demultiplexed using an in-house python script, which creates separate files for each sample by picking out the relevant primer index tag from the pooled data. TRGV and TRGJ gene assignment and CDR3 extraction was performed using an in-house python package (available on GitHub: sgp79/reptools), which assigns gene IDs by BLAST searching a database of known reads against the sequence data, then extracts CDR3 following a Smith-Waterman search to provide greater precision at the junctions.

### TRGV gene assignment

As chickens have very low germline sequence diversity in each of their TRGV gene families, ambiguously assigned sequences can occur during TRGV gene assignment. The ambiguous TRGV genes which were grouped were “TRGV2.7/2.18” and “TRGV2.4/2.8/2.14/2.19”. The majority of ambiguous gene groups account for a negligible amount of the sequence data and are excluded from analysis to enable more straightforward interpretation of the data.

### Calculation of diversity indices

Hill number diversity indices were calculated by rarefying or extrapolating to a common coverage level using the methods outlined by Colwell et al. (2012) [41]. The R package iNEXT [42] was used to calculate these indices.

### Statistical analysis

Linear models were used to provide a statistically rigorous analysis of diversity indices and TRGV gene expression levels. For diversity indices, the R base function lm() was used to fit the point estimates of diversity to tissue, or to both tissue and gene, as appropriate. For TRGV gene expression, a linear mixed effects model was built using the lmer() function of the lme4 package in R, with TRGV gene and tissue as fixed effects and individual bird as a random effect, and *p* values were calculated using the Satterthwaite approximation of degrees of freedom by the lmerTest package. In all cases, data were transformed to meet the assumptions of heteroscedasticity and normality of residuals, then back transformed for plotting [43]. When comparing the levels of publicity between different lines of bird, the ISA Browns were down-sampled to match the number of PA12 White Leghorn birds (*n* = 5).

## Results

### The chicken gamma locus shows a repeating motif suggesting tandem duplication based expansion

**Fig. 1:**
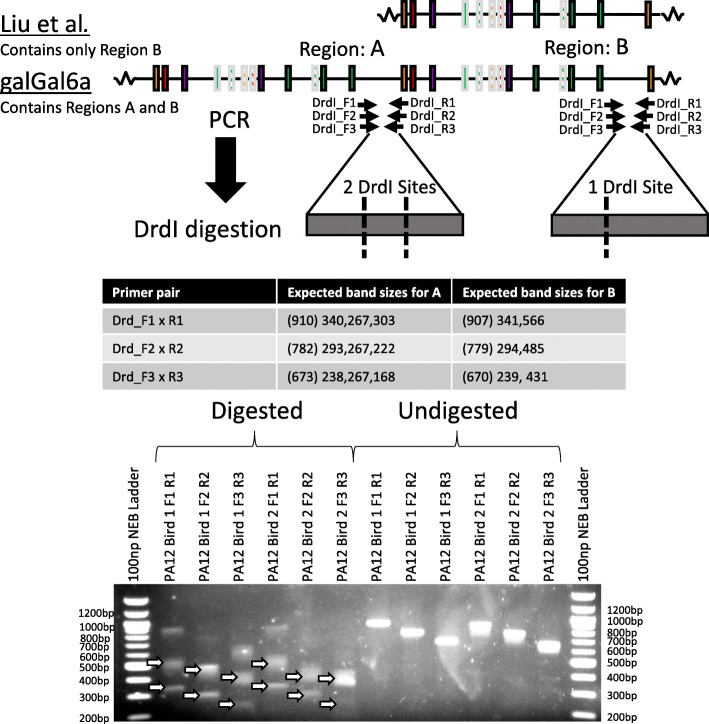
**PCR-Restriction Digest Strategy to Resolve the TCR Gamma Locus.** A schematic showing the TCR gamma locus according to Liu et al. 2020 and as depicted from galGal6a, with the latter appearing to have a tandem duplicated region. The PCR-restriction digest strategy to resolve this discrepancy is depicted including primer locations, Drdl restriction sites and expected product sizes (undigested product size in brackets). Regions of genomic DNA extracted from the spleens of 2 PA12 birds were amplified using three different primer sets and digested with the restriction enzyme Drdl and separated on a 1% agarose gel, visualised with ethidium bromide. Lanes 2–7 are with DrdI digestion, Lanes 8–13 are with no digestion. Product length was determined using a 100 bp NEB ladder. Ladder band lengths are indicated at either side of the gels in base pairs. Digested PCR products of expected sizes are indicated (white arrow) and support the Liu et al. 2020 [30] proposed structure for the TCR gamma locus

The most recent version of the chicken genome assembly, galGal6a, was supported by PacBio Single Molecule Real Time sequencing (SMRT), which produces reads with an average length of 8.5 – 14 kb. The assembly has 82x coverage, which combined with the long SMRT reads should make it a high quality and reliable whole genome sequence. However, the TCR gamma locus of this assembly was recently contradicted by Liu et al. 2020 [30] in a paper that used a BAC resequencing approach to correct an older genome assembly, galGal4. galGal6a features a 15 kb region not present in the BAC derived gamma locus [30]. To address this discrepancy, we employed a PCR and restriction endonuclease digest approach (Fig. 1). Digestion with DrdI yielded fragment sizes consistent with the 15 kb sequence being absent from the genomic DNA of PA12 White Leghorn chickens, confirming the genomic organisation proposed by Liu et al. 2020.

**Fig. 2:**
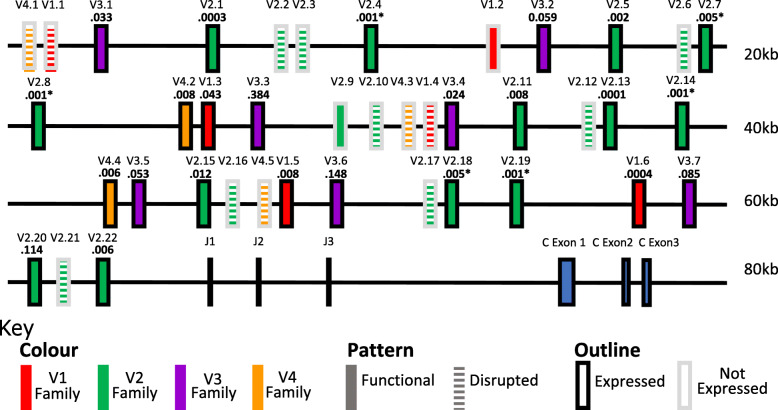
**Schematic Representation** **of the Chicken Gamma Locus**. The schematic displays the location of the coding exons for the TRGV, TRGJ and TRGC genes of TCR gamma. Four distinct families of TRGV genes (different coloured boxes) are indicated with subfamily numbering based upon genomic location. RSS sequences were identified downstream of all indicated, non-pseudogenised TRGV genes. TRGV genes with a full length ORF are indicated with a solid fill and those with a disrupted ORF by a striped fill. Those found to be expressed and rearranged by RTPCR and sequencing are bounded by a black outline and those not identified as expressed are bounded by a grey outline. The proportion of rearrangements attributed to each TRGV gene are displayed below the label. These were calculated from the averages of every tissue across every bird in this study. Genes which are too similar to be reliably distinguished from one another during the TRGV gene assignment stage of processing are represented by an asterisk

Annotation of the chicken TCR gamma locus was performed using BLAST to identify gamma-like sequences based on previously known TRGV, TRGJ and TRGC genes (Fig. 2). To provide further experimental validation of the annotation and to ensure no genes had been missed, immune repertoire derived sequence data was also mapped to the genome and BAC sequences. This revealed four TRGV genes not present in the annotation proposed by Liu et al. 2020 [30], TRGV4.1,TRGV4.3 TRGV2.12 and TRGV4.5. The TRGV gene families were classified based on their predicted Ig like domains and members within families numbered according to location 5′-3′ as proposed by IMGT. The TRGV gene families were named according to Six et al. 1996 [44] with the addition of a TRGV4 family. Forty TRGV genes were identified, grouped into four families with three TRGJs and one TRGC gene as previously reported [44]. Multiple members were identified within each TRGV family and three of the four families contained pseudogenes. The TRGV1 family contains six members (with two pseudogenes), the TRGV2 family contains twenty-two members (with eight pseudogenes), the TRGV3 family contains seven members (with no pseudogenes), and the TRGV4 family contains five members (with three pseudogenes). In the case of TRGV1.4 and TRGV2.10, the sequences of these pseudogenes have deletions compared to other family members, which could have resulted in inappropriate classification. Therefore, they were classified according to the sequence upstream of the deletion site only. Most of the intact TRGV genes were expressed at the mRNA level in thymus, spleen and intestinal tissues, with the exceptions of TRGV1.2 and TRGV2.9, which were not detected.

The addition of these new TRGV genes and their re-assignment into families offers a picture of a consistent repeating motif comprised of a TRGV4, TRGV1, TRGV3 and four TRGV2s interspersed by non-coding regions (Fig. 2). The lengths of these non-coding regions remain fairly consistent, though different iterations of this motif are subject to small differences presumably due to insertion/deletion mutations. Some of these motifs are truncated but the regions which are present still show this same characteristic structure.

**Fig. 3:**
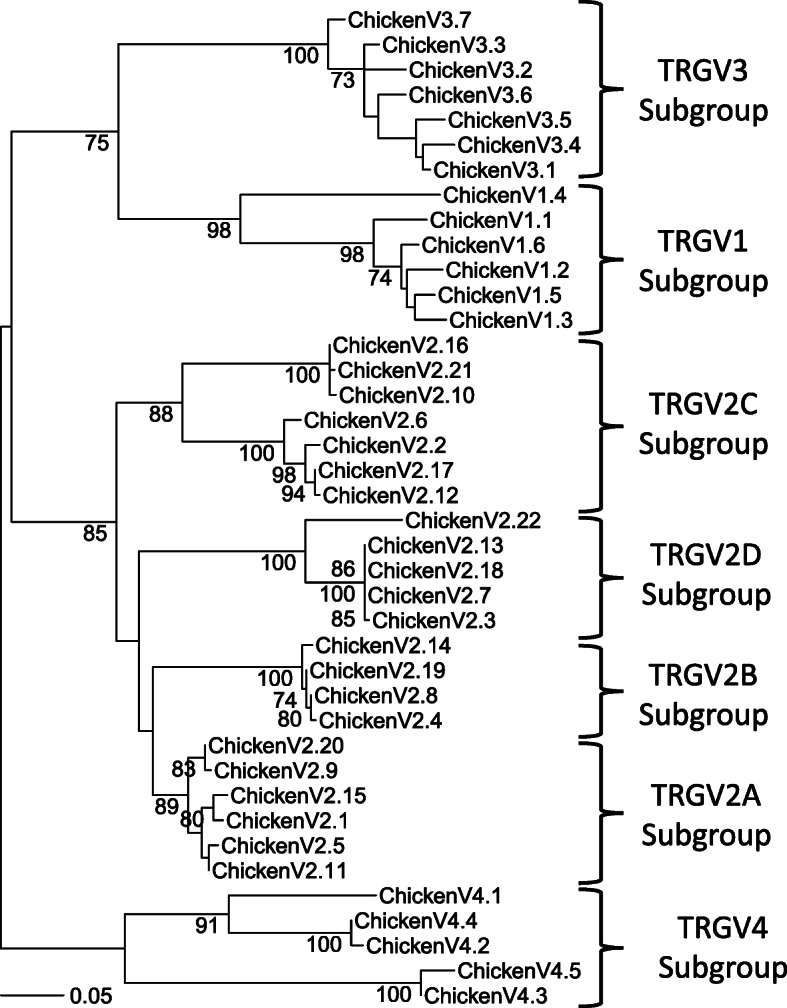
**Dotplot reveals that the Chicken TCR Gamma Locus Evolved via a set of Duplication Events.** A comparison of sequence similarity across the TCR gamma locus using a dot plot generated with Dotmatcher, window size of 1000 and threshold of 500. The default EMBOSS matrix was used (with match = + 5 and a mismatch = − 4) and a dot plotted where the value was above the threshold. The X and Y axis include schematic representations of the chicken TCR gamma locus and an indication of potentially duplicated blocks of TRGV genes. Homologous comparison is represented by the diagonal passing through the origin and the parallel diagonal indications represent regions with high similarity

**Fig. 4:**
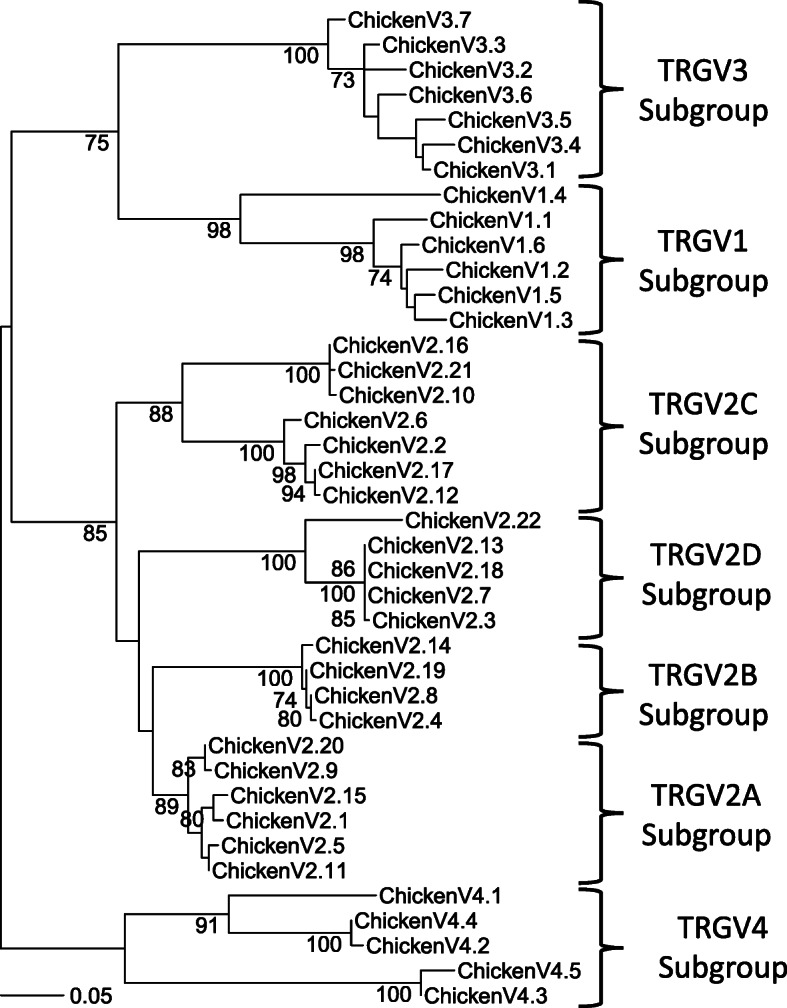
**Phylogenetic Tree of TRGV Gene Ig-like Domains.** TRGV gene Ig-like domains were aligned using MUSCLE and a phylogenetic tree calculated using maximum likelihood with a neighbour joining tree as the start point. Bootstrap values were calculated using 1000 replicates. The tree shows TRGV families clustering together in branches, which are well supported by the bootstrap values (bootstrap values > 70 are displayed). The TRGV2 genes also cluster by their position in the repeating motif, consistent with evolution by tandem duplication. Tree scale is indicated on the bottom left of the figure and shows the branch length which represents 5% of the variation

A locus-versus-locus sequence comparison revealed that the TRGV motif also showed high sequence similarity to other repeated motifs in the non-coding as well as coding regions (Fig. 3). The regions of sequence similarity between motifs are largely unbroken and often span 10-20kbp, thus strongly supporting expansion of the region by tandem duplication events. The grouping of TRGV genes into four families was also supported by a phylogenetic tree (Fig. 4). Interestingly, the TRGV2 genes clustered into four subgroups based on their position within the repeated motif (labelled A-D). The core TRGV motif therefore comprises TRGV4, TRGV1, TRGV3, TRGV2A, TRGV2B, TRGV2C and TRGV2D.

### The chicken gamma repertoire features a dominant TCR gamma rearrangement in every tissue and novel public TCRs

A 5′ RACE approach was used with multiple tissues from two lines of chicken, ISA Brown (49 days old) and PA12 White Leghorn (55 days old), to generate a broad representation of TRGV gene usage and to explore the repertoire of TCR gamma rearrangements. After amplicon sequencing and pre-processing this yielded a total of 425,870 in frame CDR3 sequences for the ISA Browns, with an average of 53,233.75 per bird and a total of 51,534 in frame CDR3 sequences for the PA12 White Leghorns, with an average of 10,306.8 per bird. To facilitate TRGV gene usage analysis, some TRGV2 genes were grouped together due to very high levels of sequence similarity.

**Fig. 5:**
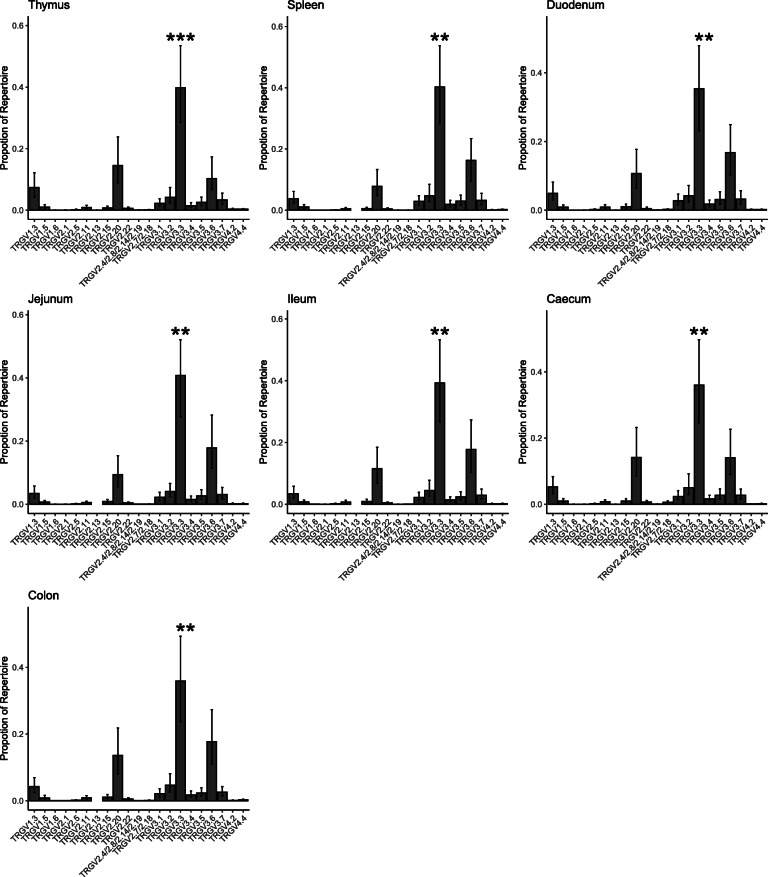
**The Proportion of each TRGV Gene Represented in TCR Gamma Chains within Different Tissues.** The proportional usage of each TRGV gene in productively (in-frame) rearranged TCR gamma is displayed in thymus, spleen and various intestinal tissues from ISA Brown chickens. Due to a high level of identity, some TRGV2 genes have been grouped. The TRGV genes on the X axis were all expressed in at least one tissue, pseudogenes and non-expressed genes are omitted. Bars represent the mean proportion for each TRGV gene displayed with 95% confidence intervals derived from 500 bootstrap replicates of an LMM using Bird number as a random variable and Tissue and TRGV gene as fixed variables. Proportions were logit transformed before model calculation. TRGV3.3 was expressed at greater levels than all other TRGV in all tissues. *P* values are comparisons between TRGV3.3 and the next most common TRGV gene in that tissue and were derived from the LMM using lmerTest with the Satterthwaite approximation of degrees of freedom. (*** *p* value < 0.001, ** *p* value < 0.01, * *p* value < 0.05)

ISA Brown birds expressed all potentially productive TRGV genes as productive rearrangements, with the exception of TRGV1.2 and TRGV2.9, although some genes were represented only as a small fraction of the sequences. The proportion of sequences attributable to each productively rearranged TRGV gene varied considerably, but the pattern was remarkably consistent between different tissues (Fig. 5). Interestingly, the profile of TRGV usage in the thymus was very close to that seen in spleen and intestinal tissues, except for a small but significant increase in the proportion of TRGV1.3 in thymus compared with jejunal and ileal tissues (*p* = 0.04 and 0.041 respectively). The mean proportion of TRGV2.20 appeared elevated in the thymic tissue, but this was variable between birds and not statistically significant (*p* > 0.05) compared with other tissues.

TRGV3.3 was the most highly expressed TRGV gene, comprising 30–40% of the entire repertoire, and this dominance persisted across all tissues (Fig. 5). TRGV3.3 was at least twice as common as the second most prevalent TRGV gene in all of the tissues, and several times the proportion of the third most prevalent. In both the TRGV1 and TRGV2 families, a single family member is expressed at much higher levels than any other, TRGV1.3 and TRGV2.20 respectively. This is not the case with the TRGV3 family, as although TRGV3.3 was more highly represented than other TRGV3 family members, all other TRGV3 family genes were well represented in sequence data derived from all tissues. The two intact members of the TRGV4 family, TRGV4.2 and TRGV4.4, were expressed at relatively low proportions and without a strong bias in expression of one over the other. It is noteworthy that the more highly expressed TRGV genes are distributed across the entire genomic region and not located in any one of the duplicated TRGV motifs.

**Fig. 6:**
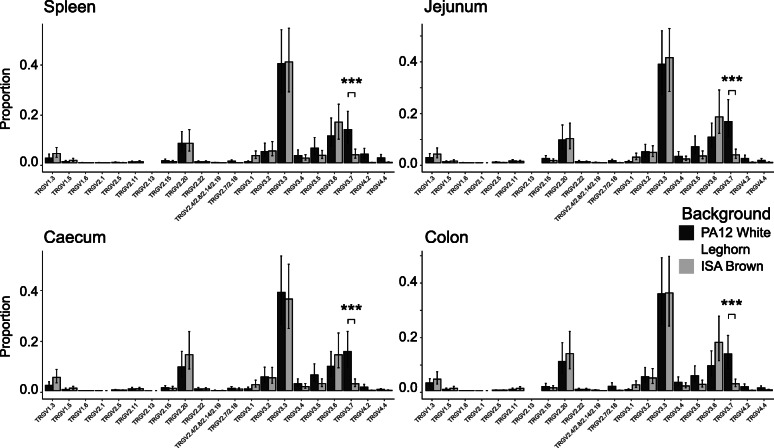
**TRGV Usage in Tissues of two Distinct lines of Chicken reveals Conserved Patterns of Expression.** The proportional usage of each TRGV gene in productively (in-frame) rearranged TCR gamma is displayed in spleen and various intestinal tissues from ISA Brown and PA12 White Leghorn lines of chicken. Due to a high level of identity, some TRGV2 genes have been grouped. The TRGV genes on the X axis were all expressed in at least one tissue, pseudogenes and non-expressed genes are omitted. Bars represent the mean proportion for each TRGV gene displayed with 95% confidence intervals derived from 500 bootstrap replicates of an LMM using Bird number as a random variable and Tissue and TRGV gene as fixed variables. Proportions were logit transformed before model calculation. *P* values were derived from the LMM using lmerTest with the Satterthwaite approximation of degrees of freedom. (*** *p* value < 0.001, ** *p* value < 0.01, * *p* value < 0.05)

Tissue samples from PA12 White Leghorn chickens were used for comparison of interline TRGV usage in spleen, jejunum, caecum and colon with the ISA Brown birds. The profiles between lines were very similar, and each included the expression of all potentially productive genes, with the exception of TRGV1.2 and TRGV2.9, although there were some small differences. TRGV1.4, TRGV3.5 and TRGV4.2 were present at a slightly higher proportion in all tissues of the PA12 White Leghorn birds, whereas TRGV1.3, TRGV3.1 and TRGV3.6 were more prominent in the ISA Browns (Fig. 6). The major difference between the two genetic backgrounds was in TRGV3.7, which represented a higher proportion of the TRGV profile in PA12 White Leghorn chickens compared with the ISA Brown birds. TRGV3.7 represented the third most prevalent TRGV gene in all tissues examined in the PA12 White Leghorn chickens, occupying 20% of all rearrangements compared with just 3% of rearrangements in the ISA Brown chickens. Both TRGV4.2 and TRGV4.4 were at higher levels in the spleen of PA12 White Leghorn chickens compared with other tissues in the PA12 background or any tissue in ISA Brown chickens.

### Private and public compartments within the chicken TCR gamma repertoire

All TCR repertoires contain a mixture of CDR3 sequences that are unique to each individual, known as the private repertoire, and CDR3 sequences that are shared between individuals, known as the public repertoire. Patterns of publicity may be important in understanding the nature and function of the T cells carrying these receptors and, in the context of γδ T cells, whether these lymphocytes might have patterns of ligand recognition consistent with a role in innate or adaptive immunity. Although definitions of publicity vary, we use it here to refer to CDR3 nucleotide sequences that are found in multiple birds in any tissue and at any proportion. These public CDR3s can be shared between several individuals or the entire population.

**Fig. 7:**
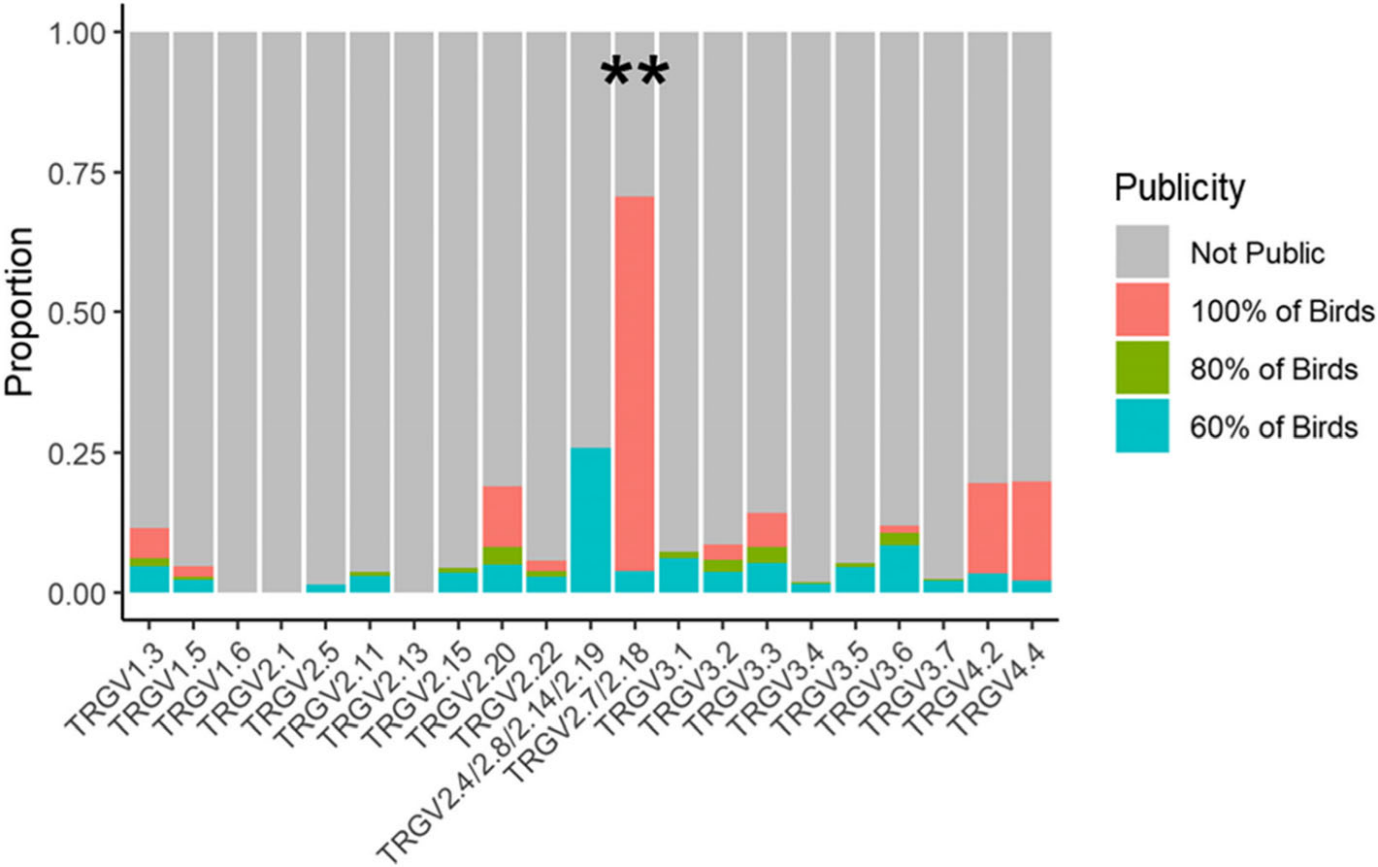
**The Proportion of Public TCR Gamma CDR3 Sequences Varies According to TRGV Gene Usage.** Publicity is defined as a CDR3 nucleotide sequence found in 60%, 80% or 100% of ISA Brown birds (*n* = 8) in any tissue at any frequency. The proportions of public clones in a given TRGV were calculated and displayed as a proportion of total sequences in the respective gene. Data is presented as stacked bars representing the proportion of the total repertoire for the particular TRGV. Due to a high level of identity, some TRGV genes have been grouped. *P* values are comparisons between the ‘100% of Birds’ category of TRGV2.7/2.18 and the next most common TRGV gene, in this case TRGV4.4, and were calculated using an unpaired Wilcoxon signed rank test. (*** *p* value < 0.001, ** *p* value < 0.01, * *p* value < 0.05)

**Fig. 8:**
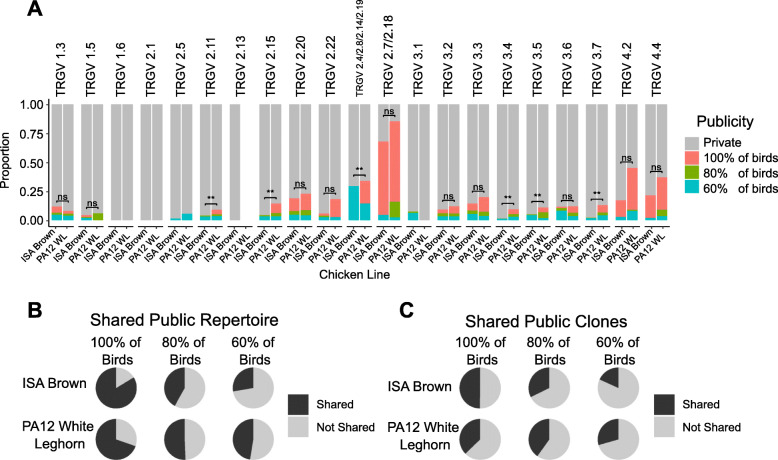
**Comparison of Publicity Profiles and Shared Public Clones between ISA Brown and PA12 White Leghorn Chickens.** **A**) Publicity was defined as a CDR3 nucleotide sequence present in 100%, 80% and 60% of birds in any tissue at any frequency in ISA Brown and PA12 chicken lines (*n* = 5/group). Publicity values were compared within TRGV genes and displayed as a proportion of all the clones within the population of TCRs defined by each TRGV. Due to a high level of identity some TRGV genes have been grouped. *P* values are comparisons between the ‘100% of Birds’ category of the two lines, and were calculated using an FDR corrected unpaired Wilcoxon signed rank test. *** *p* value < 0.001, ** p value < 0.01, * p value < 0.05. TRGV2.13 was only detected in the repertoire of the ISA Brown birds. **B**) The proportion of the overall public repertoire shared between the two lines was calculated. A public clone was considered to be shared between the two lines if it was present at the same publicity level or higher. These proportions are depicted for the 100%, 80% and 60% publicity categories for each line. **C**) The number of public clones that were shared were identified using the same definition as in B. The proportion of shared clones are depicted for the 100%, 80% and 60% publicity categories for each line

For most of the chicken TRGV genes, the CDR3 repertoire was dominated by private sequences, with 80–100% of all CDR3s only found in single individuals (Fig. 7). In the case of public CDR3 sequences, most were found in all individuals with a lower proportion exhibiting 60% or 80% penetrance in the population. The public CDR3s were represented by TCR gamma rearrangements utilising a range of TRGV genes, but publicity was much more common with some genes than others, and three genes, TRGV1.6, 2.1 and 2.13, had no public sequences at all, at a 60% publicity cut-off. By far the highest level of publicity was found in TRGV2.7/2.18 containing sequences, 75% of which were public, most often to 100% penetrance (Fig. 7). The very highly represented TRVG3.3 rearrangement was dominated by private CDR3 sequences, with public CDR3s representing ~ 10% of the entire TRVG3.3  repertoire. A similar pattern of public/private CDR3s was seen with PA12 White Leghorn chickens (Supplementary Fig. 1A, Fig. 8).

Having established that public CDR3 sequences comprise a significant proportion of the within-line repertoire in both PA12 White Leghorn and ISA Brown chickens it was important to consider the similarities and differences between these chicken lines. The proportions of public sequences within each TRGV were broadly similar between both lines although overall a slightly higher fraction of the overall repertoire was 100% public in the PA12 White Leghorn birds (Mean 0.111, SD 0.021) compared to the ISA Brown birds (Mean 0.044, SD 0.010), *p* = 0.008. Within individual TRGVs these differences were significant for TRGV2.11, 2.15, 2.4/2.8/2.14/2.19, 3.4, 3.5 and 3.7. In contrast, the TRGV specific public repertoire was larger in ISA Brown birds for TRGV1.3 and 1.5, though these differences were not statistically significant. Within TRGV2.7/2.18,the public clones dominated the repertoire in both lines of chicken. Interestingly, the public repertoire of both PA12 and ISA Brown lines of chicken was largely shared (Fig. 8B, C). For example, 70 to 80% of the public repertoire comprised identical CDR3nt sequences at the level of complete publicity (i.e. found in all birds in both lines). When considering the public clones at an equal weighting, the proportion of shared clones (Fig. 8C) was lower than when accounting for their relative contributions to the repertoire (Fig. 8B). This pattern indicates that a significant proportion of the public repertoire in both lines was composed of “expanded” or “over-represented” clones.

### Diversity of the TCR gamma repertoire

Diversity indices are metrics that can be used to compare the structure of populations, in this case clones within the TCR gamma repertoire. Since CDR3 rarefaction curves for each sample make it clear that the novel CDR3 discovery rate has not reached a plateau for any of the samples (Supplementary Fig. 2), diversity indices were calculated using rarefaction/extrapolation to a constant coverage of 0.1 [42]. Repertoire profiles were compared using Hill numbers 0, 1 and 2, which correspond to species richness, the exponential of Shannon’s entropy and the inverse of Simpson’s concentration index respectively.

**Fig. 9:**
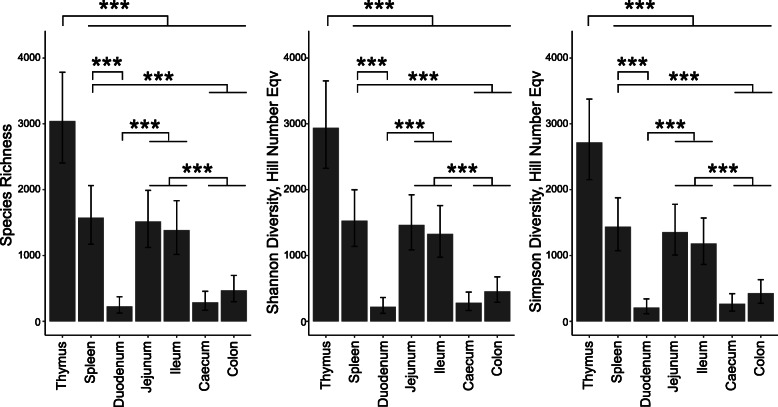
**Variation in the patterns of TCR gamma diversity in different tissues of ISA Brown chickens.** Diversity indices were calculated from sample coverage rarefied abundance data for all unique clones in a tissue using iNEXT. Diversity indices are displayed as Hill number variants and thus represent effective species counts - the number of evenly distributed species required to achieve the same diversity score. A linear model was constructed from the diversity index data using a cube root transform. Error bars are 95% confidence intervals and were extracted directly from the model along with the *P* values. Significance thresholds were as follows *** *p* value < 0.001, ** *p* value < 0.01, * *p* value < 0.05. Indications of significance are shown only for comparisons which exceeded these thresholds. The horizontal brackets indicate the groups where differences were evident and are supplemented with horizontal lines to indicate any one of multiple groups

We first considered the diversity of the global TCR gamma repertoire of ISA Brown birds according to tissue. As expected, as a site of T cell development, the thymus samples contained the most diverse repertoire, as measured by all three Hill numbers, followed by the spleen, jejunum and ileum, and then by the duodenum and large intestine (Fig. 9). In PA12 White Leghorn birds, thymus samples were not available, and lower read depth reduced the power of the analysis to the extent that it is impossible to make concrete comparisons between the other tissues, but the spleen appears to have greater diversity than the gut tissues (Supplementary Fig. 3).

**Fig. 10:**
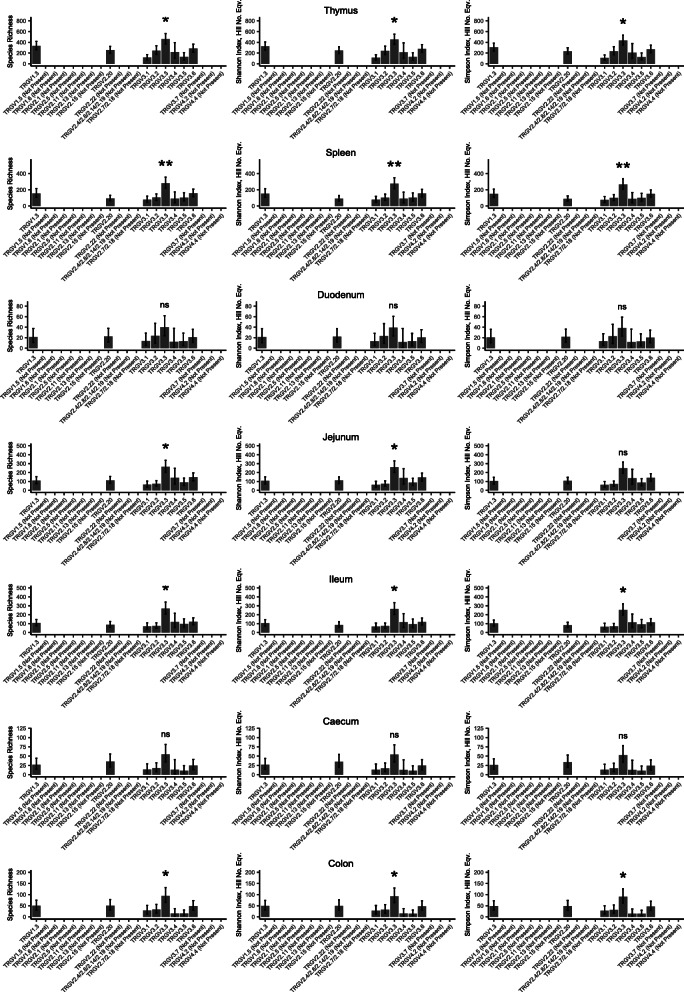
**Diversity of TRGV rearrangements in different tissues.** Diversity indices were calculated as for the aggregate ISA Brown (*n* = 8 birds) chicken tissue diversity plots using all of the clones in a TRGV gene. Due to their widely varying expression levels, genes below a read count of 100 were omitted. This overcomes the issue of rarefying down to extremely low sample coverage and therefore discarding an unacceptable amount of data. 95% confidence intervals were calculated using iNEXT. A linear model was constructed from the diversity index data using a cube root transform. Error bars are 95% confidence intervals and were extracted directly from the model along with the *P* values. P values are comparisons between TRGV3.3 and the next most diverse TRGV gene in that tissue. Significance thresholds were as follows *** *p* value < 0.001, ** *p* value < 0.01, * *p* value < 0.05

Since the overall level of expression and the proportion of public clones differed for each TRGV gene, it was important to explore whether the patterns of diversity also differed in each of these genes. These analyses were bound by the number of sequencing reads available for each gene, and therefore there was insufficient depth to meaningfully calculate diversity for the rarer genes. Of the more common genes, the clonal diversity of TRGV3.3 was higher than any other at all three Hill numbers in both ISA Brown and PA12 chickens (Fig. 10, Supplementary Fig. 4).

### The recombination signal sequence provides a partial explanation for differential TRGV gene usage

**Fig. 11:**
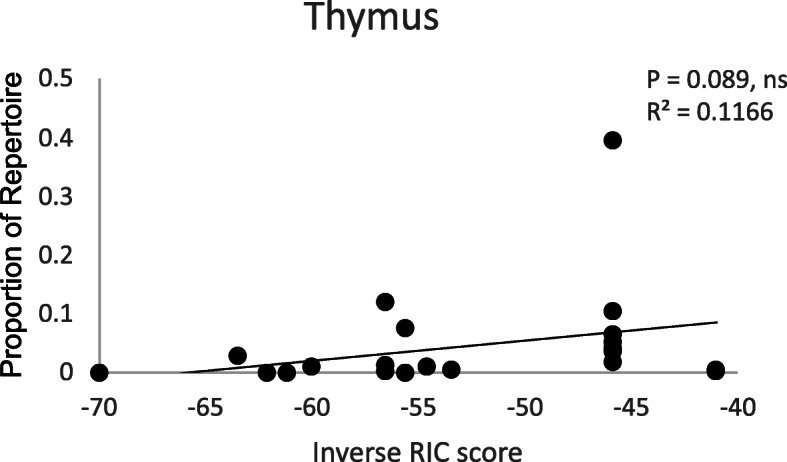
**Weak Positive  Correlation between RSS Score and TRGV Gene Expression in Thymic TCR Gamma Repertoire.** RIC scores were calculated using RSSsite and plotted against the proportion of the repertoire rearrangements attributable to each TRGV gene in the thymus of ISA Brown chickens. As there is currently no chicken RSS consensus, mouse RSS was used as a reference. These plots reveal a weak positive correlation but this only accounts for a minority of the variation we observe in the data. RIC scores are expressed as natural logs where 0 would be a perfect RSS and − 1000 would be the worst. P and R^2^ values were generated using regression analysis (*** *p* value < 0.001, ** *p* value < 0.01, * *p* value < 0.05)

Since the most highly expressed TRGVs were dispersed throughout the locus and therefore gene position was a poor indicator of TRGV gene usage, we considered whether other features, such as the Recombination Signal Sequence (RSS), might influence preferential TRGV expression. The RSS has the potential to influence the relative usage of TRGV genes and can be calculated as a Recombination Information Content (RIC) score [31]. RIC scores were calculated for each of the chicken TRGV RSSs using Bayesian models and expressed as natural logs, ranging from − 1000 to 0 where 0 would be a perfect RSS and − 1000 would represent a very poor RSS [31]. To avoid any complications associated with local peripheral tissue expansion of specific TRGVs, the proportional expression levels for each TRGV were taken from the thymus as the main site for developing T cells. The RIC scores for the TRGVs lay between − 70 and − 40 with the proportion of each TRGV ranging between < 1 and 40% of the thymic TCR gamma repertoire. Plotting the RIC scores against the proportional expression of each TRGV gene in the thymus gives a weak positive correlation with an R^2^ value of 0.1166. The *P* value from regression analysis does not quite reach a significance threshold of 0.05 (Fig. 11). The weakness of the correlation is in part due to the very high expression of TRGV3.3.

## Discussion

γδ T cells represent an enigmatic part of the immune system of all jawed vertebrates, and their retention over a prolonged period of evolution indicates that these cells play essential roles in the function of the immune system. To understand the role of γδ T cells it is critical that functions are studied in diverse animal groups with both high and low circulating levels of γδ T cells. The differences in repertoire biology are likely to relate to the potential for γδ T cells to display a spectrum of different response types, ranging from adaptive to innate [45]. To enable repertoire studies, it is important that the TCR loci in question are accurate and well annotated. Although the most recent galGal6a genome assembly has high coverage (~ 82 fold) and includes long PacBio SMRT sequencing reads, the locus organisation was different to the recent BAC resequencing based TCR gamma locus reported by Liu et al. 2020 [30]. The discrepancy was the presence of a 15 kb potential duplication in the galGal6a assembly. Our PCR restriction-digest based strategy showed that the most likely organisation of this region of the genome was indeed that proposed by Liu et al. 2020 [30]. Adding information from the repertoire sequencing revealed four further TRGV genes and showed that most intact genes were productively rearranged and expressed. The final locus map now contains 40 TRGV genes, of which 13 are pseudogenes, divided into 4 families (by 75% identity), 3 TRGJ genes and a single TRGC gene (Table 1). There was also strong phylogenetic support for the TRGV2 family to be subdivided into four sub-groups (A, B, C and D). The chicken TCR gamma locus contains a larger number of TRGV genes than any other species (Table 1) [8], although there is very little within family diversification compared with mammals [46]. It is noteworthy that chicken TRGV gene sequences are divergent from those found in mammals and do not fall into any of the conserved mammalian groups [47]. Moreover, all of the TRGVs are associated with a single TRGC as in duck [48], the only other bird that has been examined to date, and different to all of the currently resolved mammalian TCR gamma loci [47].

**Table 1:** The Number of TRGV, J and C Genes in Various Animals

***Species***	***TRGV Gene*** ***Families***	***TRGV Genes***	***TRGJ Genes***	***TRGC Genes***
Chicken	4	40 (27)	3	1
Duck	6	13 (8)	5	1
Cow	10	17 (17)	9	7
Sheep	11	13 (11)	13	6
Human	6	15 (6)	5	2
Mouse	5	7 (7)	4	4

Numbers of TRGV, TRGJ and TRGC genes in the chicken compared with those of duck [48], cow, sheep, human, and mouse [36]. TRGV gene families were grouped according to IMGT subgroup concept of 75% sequence identity at the nucleotide level. Total numbers of TRGV genes are shown, with the number of functional TRGV genes, not including ORFs, shown in brackets. The mouse TCR gamma locus is based on multiple cassettes and one mouse TRGC is a pseudogene and therefore one TRGV gene is not used.

Confirmation of the TCR gamma locus sequence, the discovery of four additional TRGVs, and the assignment of gene families and the TRGV2 subfamilies collectively, revealed a pattern that suggests a block-based expansion of the locus by tandem duplication. Further evidence for this hypothesis is provided by the dot plot which shows multiple regions of high sequence similarity dispersed throughout the locus. The patterns of similarity include both the TRGV motif patterns and extensive noncoding regions, providing strong evidence for tandem duplication. Duplication based expansion of TCR loci is widely reported with other animals [46] and the very high sequence similarity in non-coding and coding regions supports the premise that these represent recent events. The lengths of most intergenic regions are consistent and many of the differences can be identified as partial block duplication or deletion events. This extensive duplication suggests recent strong selective pressure for diversification of the TCR gamma region as reported for the TCR and other loci [46, 49, 50]. The level of identity within chicken TRGV family members is much higher than can be seen with other animals [46] which also supports the hypothesis of a recent duplication event. One driver for duplication might be to expand the repertoire of the γδ T cell population, however, the very high identity of some TRGV and the pseudogenisation of a number of others are not consistent with this hypothesis, though this may simply be due to a lack of time, which would allow diversification of function to develop. The motif most distal from the TRGC gene is likely to be the ancestral region as it possesses the longest array of duplicated TRGV genes with the most highly conserved gap sizes, though this is speculative. Moreover, the repertoire analysis shows that a single TRGV dominates the repertoire within each family with other family members being represented at very low levels. This suggests a complex array of selective pressures expanding the region then limiting the use of the duplicated TRGVs.

In terms of explaining the differential usage of TRGV genes, the RSS RIC score has some predictive value but only explains a small amount of the variation in thymic TRGV usage. Other features such as gene order can influence TRGV expression, particularly during foetal development [51]. However, this did not correlate with proportional expression of TRGV in the chicken thymus or other tissues. A more likely explanation is that usage may also be affected by promoter sequence or modifications such as methylation [51–53].

The similarity of TRGV usage profiles between tissues was surprising since in both humans and mice there is a significant bias in TRGV usage in different tissues. In particular, with the foetal-thymus derived epithelial-associated subsets of γδ T cells in the mouse, TRGV5, 6 and 7, home preferentially to different sites within the body [54, 55]. Similarly, TRGV9 expressing γδ T cells predominate in the blood of humans whereas TRGV4 is most common in the intestine [56]. At least some of these patterns of TRGV bias are influenced by epithelial expression of selective agents such as the butyrophilin-like molecules [56]. The influence of these types of molecule on TRGV repertoire remains undefined in the chicken, or any other γδ high animal, although the lack of a strong tissue-associated bias in chicken TRGV expression would need to be considered in such studies.

In terms of the repertoire of chicken γδ T cells, this population contains a high diversity of CDR3s, especially within the dominantly expressed TRGV genes such as TRGV3.3. A large proportion of this diversity was represented as private CDR3 sequences. That said, there were also substantial fractions of the overall repertoire that can be considered highly public and were found in a high proportion, often 100%, of the birds in this study. Moreover, the proportion of these hyper-public CDR3s varied between TCR gamma rearrangements that utilised different TRGV genes and the patterns of publicity were remarkably similar between PA12 and ISA Brown lines of chicken. The biology of these public versus private repertoires could be critical in defining the function of chicken γδ T cells. Indeed, the skin and uterine/tongue focussed murine γδ T cells represent hyper-public canonically rearranged versions of TRGV5 and TRGV6 respectively [16]. The Vγ5Vδ1+ DETCs in mice have been shown to play important roles in tumour surveillance, inflammation and epithelial cell regulation [19, 20]. The Vγ9 + Vδ2+ T cells found in the blood of humans are a semi-invariant population of γδ T cells where approximately 80% of clones contain public CDR3s [57, 58]. Interestingly, these Vγ9 + Vδ2+ T cells have been shown to respond to pyrophosphate antigens and are activated during infection with pathogens such as *M. tuberculosis* [59]*.* Highly public CDR3s were detected in chicken TCR gamma, and the clones which were present in 100% of birds made up over 70% of the rearrangements expressing TRGV2.7/2.18, which suggests a specialised function for cells bearing this receptor. Moreover, many of the public clones, and their proportions within the repertoire, were shared between two genetically distinct lines of chicken indicating the ubiquity of the public TRGV repertoire. The sub-division of γδ T cells according to publicity could be important in understanding the spectrum of roles fulfilled by γδ T cells in different species.

The diversity of TCR gamma in the chicken is highest in the thymus, which is in line with expectations, as this is the primary site where γδ T cell maturation occurs [60], and would therefore potentially contain all the TCR gamma rearrangements that exist in the chicken, though some debate remains about the existence and function of extrathymically derived γδ T cells in some species [60–62]. On the other hand, the large intestinal tissues, caecum and colon, being less diverse than jejunum and ileum is contrary to our expectations. Considering the role of γδ T cells in the early response to pathogens at epithelial sites [16], we might expect to see a greater receptor diversity in the caecum and colon as these tissues host the major microbial population in the gut and therefore the greatest “antigenic” diversity. This result potentially implies that γδ T cells may be more important in responses to microbes in the small intestine, or that they are carrying out some other function in this part of the gut that requires greater receptor diversity. Furthermore, we showed that TRGV gene diversity correlated with proportional expression, therefore we conclude that the TRGV genes with the highest diversity are rearranged preferentially. The results presented here represent the state of the “mature” TCR gamma repertoire, as the birds examined were all 7–8 weeks of age, and in a period of stable γδ T cell export from the thymus, about 4 weeks after the last of the three “waves of thymic γδ T cell emigration” [63] . It will be of interest to consider the dynamics of TRGV repertoire patterns in younger birds during the pre-hatch and early post hatch periods.

## Conclusion

The regional differences in repertoire may reflect the numbers of γδ T cells in these different tissues and could influence how different tissue resident populations of γδ T cells interact with the outside world, including pathogens and the microbiota. The TRGV repertoire analyses used here represent an important tool for use in future studies and have been critical in understanding the TCR gamma locus in chickens. This work is a starting point for understanding the role for γδ T cells in chickens and more broadly in γδ high animals that may be more reliant on these cells than the more commonly studied γδ low animals. A broad understanding of γδ T cell biology and the driving forces that have underpinned their retention across the jawed vertebrates will help us understand the evolution of immune capabilities and may reveal new ways for us to exploit these cells to protect humans and livestock species.

## Supplementary Information

**Additional file 1.** Primers Used in this Study

**Additional file 2: Supplementary Fig. 1.** - Levels of Publicity in each TRGV Gene in PA12 White Leghorn Chickens. **Supplementary Fig. 2.** - Rarefaction Plots generated using iNEXT. **Supplementary Fig. 3.** - Variation in the Patterns of TRGV Gamma Diversity in Tissues of PA12 White Leghorn Chickens. **Supplementary Fig. 4.** - Diversity of TRGV Genes in different Tissues

## Data Availability

The datasets and aliquots of remaining materials generated during the current study are available from the corresponding author on reasonable request. Datasets are also available at the European Nucleotide Database (https://www.ebi.ac.uk/ena/browser/home) Accession number PRJEB44890.
