# Correction to: Repertoire analysis of γδ T cells in the chicken enables functional annotation of the genomic region revealing highly variable pan-tissue TCR gamma V gene usage as well as identifying public and private repertoires

**DOI:** 10.1186/s12864-021-08128-6

**Published:** 2021-11-16

**Authors:** Robert Dixon, Stephen G. Preston, Stefan Dascalu, Patrik G. Flammer, Steven R. Fiddaman, Kirstie McLoughlin, Amy Boyd, Jiri Volf, Ivan Rychlik, Michael B. Bonsall, Bernd Kaspers, Adrian L. Smith

**Affiliations:** 1 Department of Zoology, University of Oxford, Oxford, UK; 2 The Pirbright Institute, Ash Road, Pirbright, Woking, Surrey, UK; 3 Veterinary Research Institute, Brno, Czech Republic; 4 Veterinary Faculty, Ludwig Maximillians University, Planegg, Germany

**Correction to: BMC Genomics 22, 719 (2021)**

**https://doi.org/10.1186/s12864-021-08036-9**

Following publication of the original article [1], the authors identified an error in Fig. 3 and 6. In addition one of the affiliations was incorrectly assigned. The correct figures are given hereafter and the affiliation change has been implemented.

**Fig. 3:**
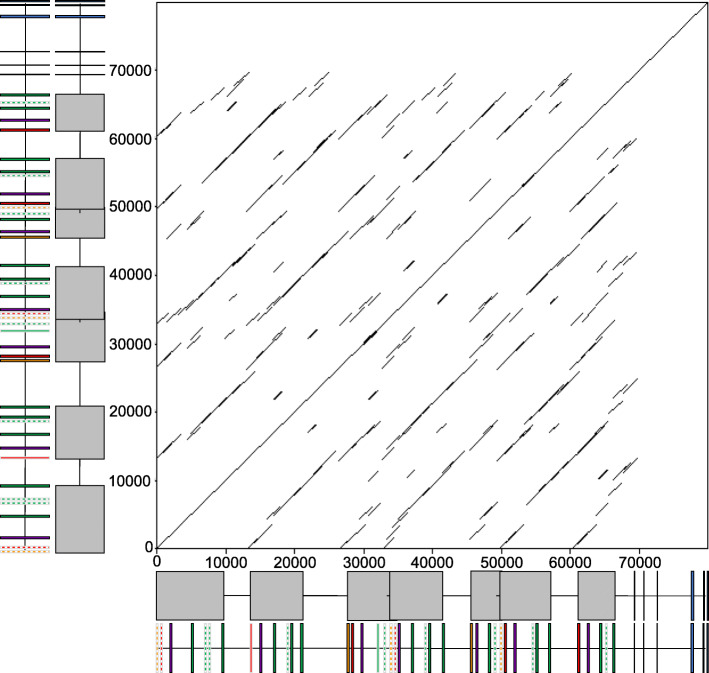
Dotplot reveals that the Chicken TCR Gamma Locus Evolved via a set of Duplication Events. A comparison of sequence similarity across the TCR gamma locus using a dot plot generated with Dotmatcher, window size of 1000 and threshold of 500. The default EMBOSS matrix was used (with match = + 5 and a mismatch = − 4) and a dot plotted where the value was above the threshold. The X and Y axis include schematic representations of the chicken TCR gamma locus and an indication of potentially duplicated blocks of TRGV genes. Homologous comparison is represented by the diagonal passing through the origin and the parallel diagonal indications represent regions with high similarity hide

**Fig. 6:**
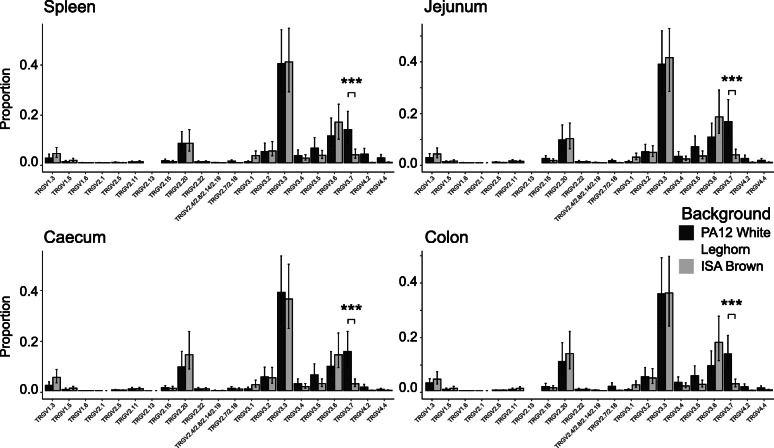
TRGV Usage in Tissues of two Distinct lines of Chicken reveals Conserved Patterns of Expression. The proportional usage of each TRGV gene in productively (in-frame) rearranged TCR gamma is displayed in spleen and various intestinal tissues from ISA Brown and PA12 White Leghorn lines of chicken. Due to a high level of identity, some TRGV2 genes have been grouped. The TRGV genes on the X axis were all expressed in at least one tissue, pseudogenes and non-expressed genes are omitted. Bars represent the mean proportion for each TRGV gene displayed with 95% confidence intervals derived from 500 bootstrap replicates of an LMM using Bird number as a random variable and Tissue and TRGV gene as fixed variables. Proportions were logit transformed before model calculation. *P* values were derived from the LMM using lmerTest with the Satterthwaite approximation of degrees of freedom. (*** *p* value < 0.001, ** *p* value < 0.01, * *p* value < 0.05)

All the changes requested are implemented in this correction and the original article [1] has been corrected.
