# Supplementary material for: Repertoire analysis of γδ T cells in the chicken enables functional annotation of the genomic region revealing highly variable pan-tissue TCR gamma V gene usage as well as identifying public and private repertoires
Source: BMC Genomics. 2021 Oct 6;22:719. doi: 10.1186/s12864-021-08036-9 (PMC8493715; doi:10.1186/s12864-021-08036-9)
Supplement: Supplementary file 1 — Additional file 1. Primers Used in this Study [file 12864_2021_8036_MOESM1_ESM.docx]

# Primers Used in this Study

| **Primer** | **Sequence (5’-3’)** |
| --- | --- |
| DrdI_F1 | CAGAGAAAGGCAGAGCTCC |
| DrdI_F2 | GGTTTTCTGGGCAATGCAGT |
| DrdI_F3 | TTAAGAGCGCAGTACCAACAC |
| DrdI_R1 | CCAGTAAGCACAGTCATAGGTC |
| DrdI_R2 | GTCTGTAGCCAAGTTGTGTGC |
| DrdI_R3 | CTGTTTGTAACGTCCTGCCC |
| UPA short | CTAATACGACTCACTATAGGGC |
| UPA long | CTAATACGACTCACTATAGGGCAAGCAGTGGTATCAACGCAGAGT |
| Barcoded Chicken TCR gamma* | GGTAGAATTTCTCAATAAGACAAACATACACCACTT |

*7bp barcode sequences, generated according to Bystrykh 2012^40^, were attached during synthesis to the start of each primer and a different one used for each sample to allow sequences obtained during downstream NGS to be assigned back to their sample of origin.
