# Supplementary material for: Repertoire analysis of γδ T cells in the chicken enables functional annotation of the genomic region revealing highly variable pan-tissue TCR gamma V gene usage as well as identifying public and private repertoires
Source: BMC Genomics. 2021 Oct 6;22:719. doi: 10.1186/s12864-021-08036-9 (PMC8493715; doi:10.1186/s12864-021-08036-9)
Supplement: Supplementary file 2 — Additional file 2: Supplementary Fig. 1. - Levels of Publicity in each TRGV Gene in PA12 White Leghorn Chickens. Supplementary Fig. 2. - Rarefaction Plots generated using iNEXT. Supplementary Fig. 3. - Variation in the Patterns of TRGV Gamma Diversity in Tissues of PA12 White Leghorn Chickens. Supplementary Fig. 4. - Diversity of TRGV Genes in different Tissues [file 12864_2021_8036_MOESM2_ESM.docx]

# Supplementary Figures


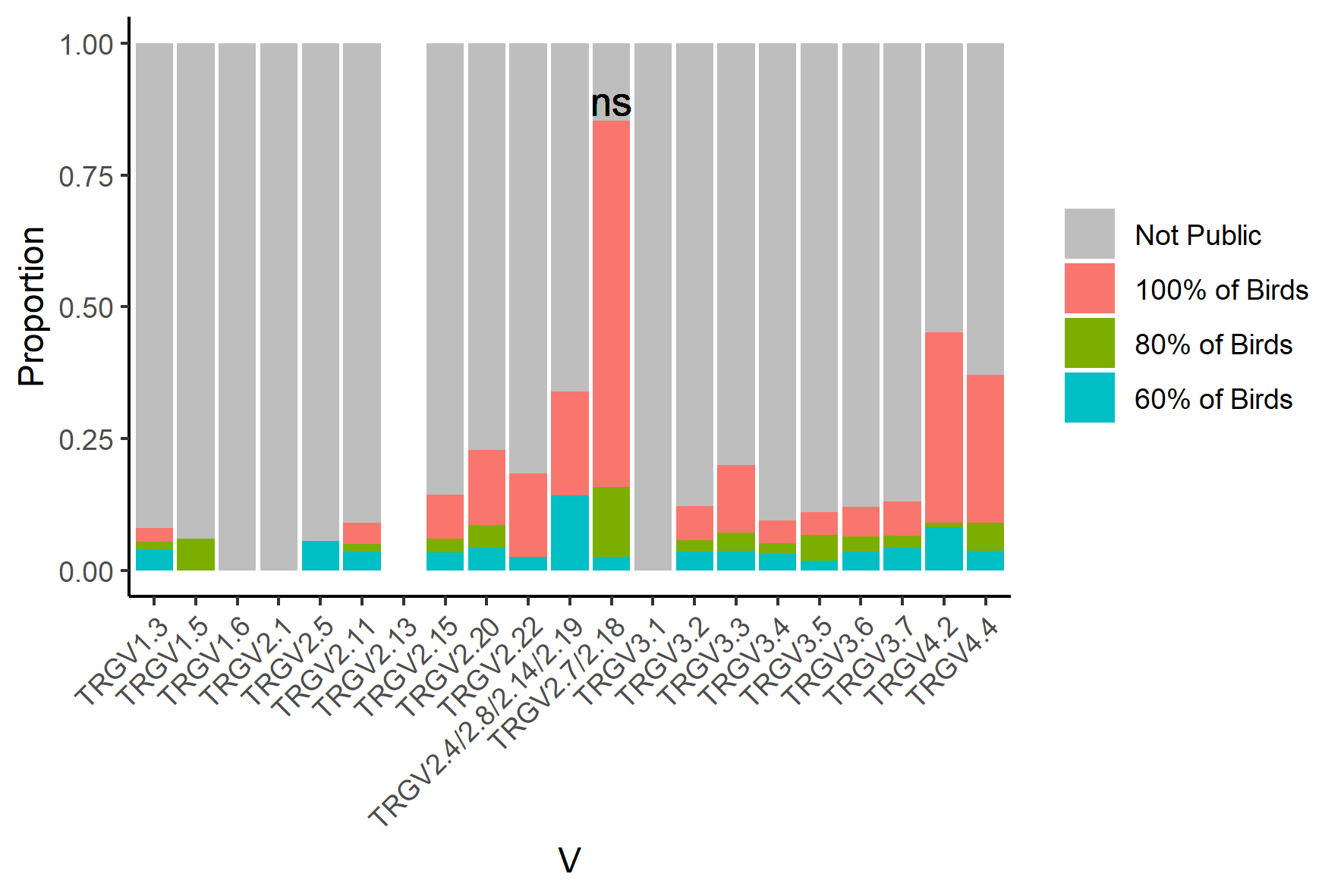


**Supplementary Figure 1 - Levels of Publicity in each TRGV Gene in PA12 White Leghorn Chickens**

Publicity is defined as a CDR3 nucleotide sequence found in 60%, 80% or 100% of birds (n=5) in any tissue at any frequency. The proportions of public clones in a given TRGV were calculated and displayed as a proportion of total sequences in the respective TRGV. Data is presented as stacked bars representing the proportion of the total repertoire for the particular TCRV. Due to a high level of identity, some TRGV genes have been grouped. P values are comparisons between the ‘100% of Birds’ category of TRGV2.7/2.18 and the next most common TRGV gene, in this case TRGV4.2, and were calculated using an unpaired Wilcoxon signed rank test. *** p value < 0.001, ** p value < 0.01, * p value < 0.05.


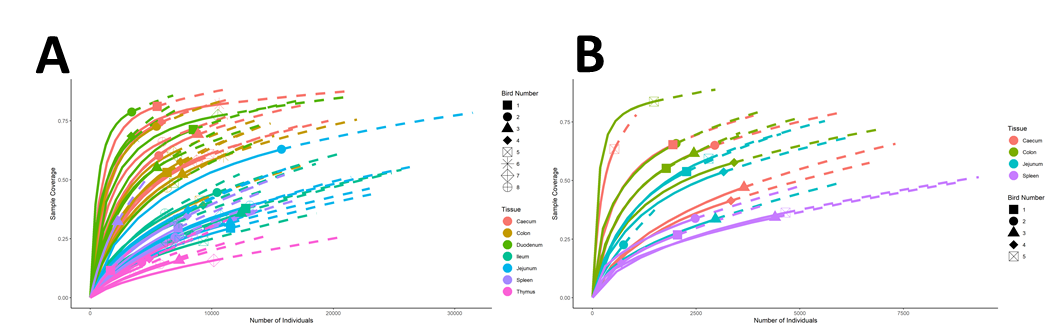


**Supplementary Figure 2 - Rarefaction Plots generated using iNEXT**

Plots show rarefaction curves of numbers of individuals, in this case represented by read number, against sample coverage for the **A)** ISA Brown and **B)** PA12 chicken lines. Plots were generated using iNEXT and as the number of individuals sampled increases, the coverage also increases but at a diminishing rate. Solid lines represent interpolated data, whereas dashed lines are extrapolations up to 2x the read number of the original sample. Coverage is calculated using Chao coverage. Line colour represents tissue, and the point shape, which is plotted at the intersection between the actual number of sequences and coverage of any given sample, represents bird number.


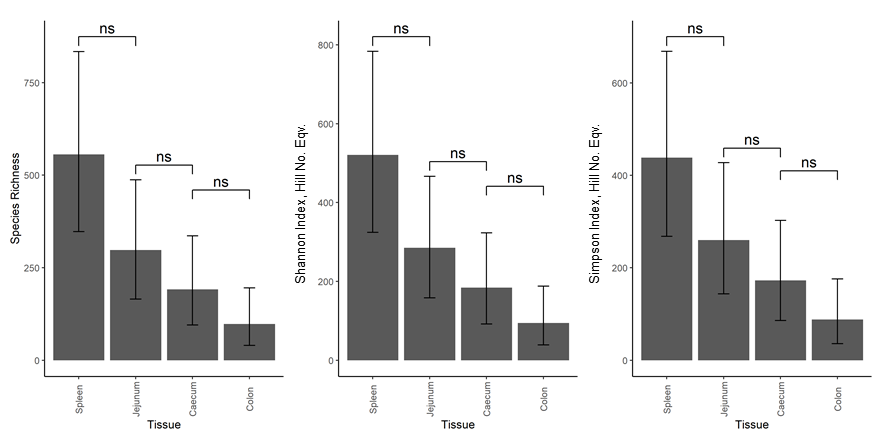


**Supplementary Figure 3 - Variation in the Patterns of TRGV Gamma Diversity in Tissues of PA12 White Leghorn Chickens**

Diversity indices were calculated from sample coverage rarefied abundance data for all clones in a tissue using the iNEXT platform. Diversity indices are displayed as Hill number variants and represent effective species counts - the number of evenly distributed species required to achieve the same diversity score. Indices are ordered by increasing Hill number and thus decreasing sensitivity to rare species. 95% confidence intervals were calculated using iNEXT. A linear model was constructed from the diversity index data using a cube root transform. Error bars are 95% confidence intervals and were extracted directly from the model along with the P values. Significance thresholds were as follows *** p value < 0.001, ** p value < 0.01, * p value < 0.05.


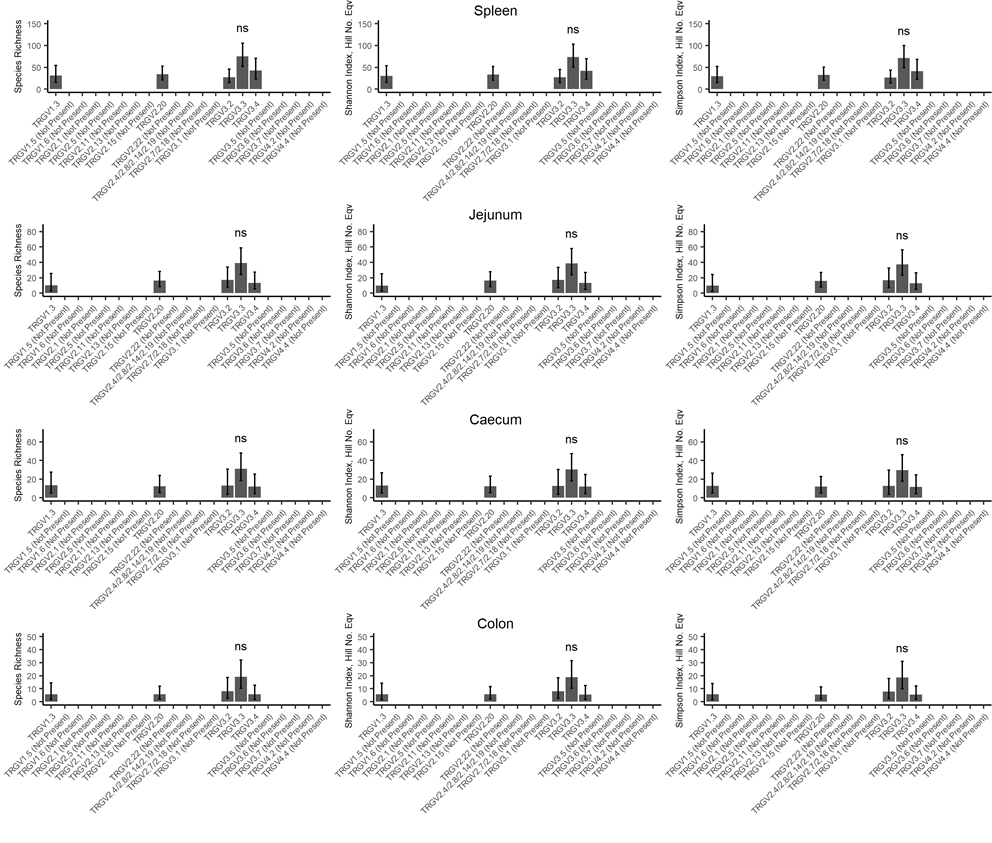


**Supplementary Figure 4 - Diversity of TRGV Genes in different Tissues**

Diversity indices were calculated as for the aggregate tissue diversity plots using all of the clones in a TRGV gene. Due to their widely varying expression levels, genes below a read count of 100 were omitted. This overcomes the issue of rarefying down to extremely low sample coverage and therefore discarding an unacceptable amount of data. 95% confidence intervals were calculated using iNEXT. A linear model was constructed from the diversity index data using a cube root transform. Error bars are 95% confidence intervals and were extracted directly from the model along with the P values. P values are comparisons between TRGV3.3 and the next most common TRGV gene in that tissue Significance thresholds were as follows *** p value < 0.001, ** p value < 0.01, * p value < 0.05.
